# Evaluation of Endothelial Dysfunction in Geriatric Patients with Non-Dialysis Chronic Kidney Disease

**DOI:** 10.3390/jcm15124708

**Published:** 2026-06-17

**Authors:** Alper Alp, Irmak Taşkıran Uyar, Zeynep Filiz Eren, Melike Ersoy, Ercan Saruhan, Dilek Gibyeli Genek, Bülent Huddam

**Affiliations:** 1 Department of Nephrology, Faculty of Medicine, Mugla Sitki Kocman University, Mugla 48000, Turkey; 2 İç Hastalıkları Kliniği, Aydın Atatürk Devlet Hastanesi, Aydın 09020, Turkey; 3 Faculty of Computer Engineering, Mugla Sitki Kocman University, Mugla 48000, Turkey; 4 Department of Rheumatology, Faculty of Medicine, Mugla Sitki Kocman University, Mugla 48000, Turkey; 5 Department of Biochemistry, Faculty of Medicine, Mugla Sitki Kocman University, Mugla 48000, Turkey; ercansaruhan@mu.edu.tr

**Keywords:** chronic kidney disease, geriatrics, soluble VE cadherin, carotis intima media thickness, nailfold capillaroscopy, FGF-23, flow-mediated dilation

## Abstract

**Background**: Chronic kidney disease presents a significant health challenge among the elderly, with recent data indicating a 13.9% prevalence for early stages (1–3) and a lower 0.6% prevalence for advanced stages. Notably, many geriatric patients die from cardiovascular complications before reaching end-stage kidney disease, highlighting the critical interplay between renal and cardiovascular health. Central to this connection is endothelial dysfunction, considered the initial trigger for cardiovascular mortality. We aimed to investigate the correlation between different measurement methods demonstrating endothelial dysfunction and sVE-cadherin levels. Another objective was to examine the relationship between decreased glomerular filtration rate (GFR) and sVE-cadherin levels. We hypothesized an inverse relationship between impaired renal function, endothelial dysfunction, and sVE-cadherin. **Methods**: The study included geriatric patients with CKD who were not receiving RRT. Non-geriatric patients, those with cardiovascular disease, atrial fibrillation, heart failure, active immunosuppressive use, active infection, history of active malignancy, Raynaud’s phenomenon, and renal transplantation patients were excluded. Demographic data of the patients, nailfold capillary measurements, carotid intima-media thickness, flow-mediated dilatation, sVE-cadherin, and serum fibroblast growth factor 23 (FGF23) levels were measured. **Results**: We analyzed 96 patients. Key findings revealed a significant inverse correlation between serum sVE-cadherin levels and glomerular filtration rate (GFR), suggesting that, as kidney function declines, endothelial integrity is compromised. Interestingly, patients treated with sodium–glucose co-transporter-2 inhibitors had notably lower sVE-cadherin levels, indicating the possible modulatory effect of these drugs on endothelial function. Additional correlations were observed: fibroblast growth factor 23 levels were positively related to capillary diameter, and carotid intima-media thickness was associated with mean platelet volume. Declining GFR corresponded to reductions in capillary count, while use of dipeptidyl peptidase-4 inhibitors was linked to higher capillary density. Over a 2.3-year follow-up, survivors had higher lymphocyte counts (*p* = 0.088, not statistically significant) and baseline sVE-cadherin levels tended to be higher in those who died, although this was not statistically significant. **Conclusions**: These findings suggest that uremic toxins may worsen endothelial injury by disrupting intercellular connections, highlighting the complex pathogenic environment in CKD. Given these insights, the need for standardized diagnostic thresholds for endothelial dysfunction in geriatric CKD patients is clear. Serum sVE-cadherin emerges as a promising novel biomarker for assessing endothelial health, offering potential for earlier intervention and improved cardiovascular outcomes. It may be a potent indicator of endothelial dysfunction and should be featured in future studies of elderly CKD patients.

## 1. Introduction

Chronic kidney disease (CKD) is a global health problem that is becoming increasingly prevalent worldwide; it is one of the few non-communicable diseases with increasing mortality rates. The global age-standardized prevalence of CKD in adults is 14.2%; CKD is among the fastest-growing causes of death worldwide. The global age-standardized mortality rate due to CKD is estimated at 26.5 (23.1–29.5) deaths per 100,000 in 2023. It is projected to become the fifth leading cause of disability-adjusted life-years (DALYs) by 2040. Globally, it is estimated that one in five men and one in four women aged 65–74 have CKD. Most patients with CKD pass away before reaching end-stage renal failure due to cardiovascular disease (most common), acute kidney injury (AKI), and infections. In 2023, 11.5% (95% uncertainty interval [UI]: 8.4–14.5) of global deaths from cardiovascular disease were attributed to renal dysfunction. The global age-standardized prevalence of CKD not requiring renal replacement therapy (RRT) is 9.0% (95% UI: 8.3–9.9) for stages 1–2 and 4.5% (4.2–4.9) for stage 3 [1]. Therefore, strategies for reducing mortality in geriatric patients with CKD who are not receiving RRT but are at risk of cardiovascular disease are the most prioritized targeted approaches to managing this major global disease and its cost burden.

In patients with CKD, subclinical abnormalities in microcirculation are recognized as a predisposition to long-term cardiovascular disease. A proactive approach to cardiovascular disease is crucial for reducing mortality. Early ED detection is the primary goal of this approach using indirect methods. ED leads to angiogenic defects, vascular inflammation, increased vascular resistance, and decreased tissue perfusion, resulting in organ dysfunction. Microvascular dysfunction in the kidneys leads to significant changes in glomerular endothelial cells, such as decreased capillary density and endothelial cell loss, which can result in a progressive reduction in renal function.

Regarding the inflammatory microenvironment in patients with CKD, adverse structural changes occur, such as disruption in tight and adherence junctions connecting adjacent endothelial cells, increased paracellular permeability, and impaired barrier function, leading to fluid extravasation, loss of microcirculatory flow, angiogenic defects, and increased pro-inflammatory and prothrombotic status. In addition to these changes, which can be observed in both peripheral and coronary microvascular structures, a decrease in structural and functional capillary density is observed as CKD progresses.

### 1.1. VE-Cadherin/sVE-Cadherin

Endothelial intercellular junctions facilitate the transfer of intercellular signals, playing a role in inhibiting cell growth, protecting against apoptosis, regulating cell polarity, and migration. The endothelial barrier comprises tight and adherence junctions. At adherence junctions, adhesion occurs via vascular endothelial cadherin (VE-cadherin). VE-cadherin, an endothelial transmembrane glycoprotein, is the primary intercellular adhesion molecule that forms adherence junctions and ensures their integrity. VE-cadherin binds directly to β-catenin, plakoglobulin, and p120 intracellularly. It plays an important role in angiogenesis (by regulating activity of vascular endothelial growth factor receptors), maintaining vascular integrity and permeability via the endothelial barrier. Ca^2+^-dependent VE-cadherin also has a function in the transmission of a wide variety of extracellular signals that regulate cell growth and polarity, lumen formation, and responses to shear stress [2]. VE-cadherin, which is specific to endothelial cells, contributes significantly to inflammation by regulating paracellular extravasation of leukocytes.

The influx of Ca^2+^ into cells is a signal of damage; it confers a prothrombotic and pro-inflammatory phenotype in endothelial cells, causing VE-cadherin degradation. This leads to increased vascular permeability. Activation of the Src signaling pathway results in VE-cadherin phosphorylation and endocytosis, destruction of adherens junctions, and increased endothelial permeability [3]. VE-cadherin is degraded under hypoxic and inflammatory conditions (via neutrophil elastase, cathepsin G, disintegrin, and the metalloproteinase ADAM10), leading to the release of sVE-cadherin fragments predominantly consisting of endothelial cell 1–5 domains. Increased levels of sVE-cadherin fragments are considered biomarkers of damage to the endothelial adherens junction [4]. Disruption in the resulting adherens junction leads to increased vascular permeability, facilitating the migration of immune cells from the circulation. However, it has also been noted that circulating sVE-cadherin has a destructive effect on the endothelial barrier and an anti-angiogenic effect.

Transdifferentiation, characterized by the progressive loss of morphological, molecular, and functional phenotypes of endothelial cells and their simultaneous transformation into a profibrotic mesenchymal/myofibroblast-like profile, is known as endothelial-to-mesenchymal transition (EndMT). During EndMT, endothelial cells undergo morphological differentiation through cytoskeletal remodeling, gain the ability to migrate or contract, and undergo an immunophenotypic change characterized by the loss of endothelium-specific markers, such as VE-cadherin [5]. Endothelial cells are exposed to EndMT when stimulated by oxidized low-density lipoprotein, oxidative stress, hypoxia, shear stress, and inflammation, leading to the structural degradation of VE-cadherin and the breakdown of adherens junctions. Reactive oxygen species (ROS) play a significant role in regulating vascular function and can cause endothelial barrier dysfunction. Increased ROS production promotes internalization and degradation of VE-cadherin. Depletion of VE-cadherin leads to impaired endothelial integrity/increased permeability (infiltration of pro-atherogenic molecules/inflammatory cells into the vessel wall), intermittent hypoxia, increased atheroma plaque calcification, thinning of the fibrous valve, and plaque instability [6].

The cardiovascular risk increases in patients with microvascular dysfunction, leading to tissue hypoxia. A 2004 study found that the sVE-cadherin concentration was higher in patients with atherosclerotic heart disease than in healthy individuals, independent of other risk factors; this has been interpreted as an indicator of a cardiac hypoxic microenvironment [7].

### 1.2. Fibroblast Growth Factor-23 (FGF23)

FGF23 is a phosphotropic hormone from the fibroblast growth factor family. Nephrosclerosis, commonly observed in patients with CKD, is caused by systemic vascular endothelial dysfunction in the renal vascular endothelial structure. Inflammation, oxidative stress, hyperphosphatemia and low serum vitamin D, high FGF23, and low Klotho levels are the main risk factors of vascular dysfunction in patients with CKD [8]. High serum FGF23 levels are known to be independent risk factors for vascular dysfunction, left ventricular hypertrophy, increased risk of end-stage renal disease, and mortality in patients with CKD [9].

### 1.3. Carotid Intima-Media Thickness (CIMT)

CIMT is a measurement of the distance between the carotid artery lumen intima and medial adventitial surfaces. CIMT indicates the presence and progression of atherosclerosis and is a marker of structural arterial diseases. Since CIMT is a simple, repeatable, and non-invasive measurement, it is widely used to identify cardiovascular risk [10]. Prospective studies have reported strong associations between CIMT and myocardial infarction, stroke, CVD-related death, or a combination of these factors [11]. In addition, increased CIMT levels have been observed in patients with advanced CKD, those receiving renal replacement therapy, and recipients of renal transplants [12].

### 1.4. Flow-Mediated Dilatation (FMD)

The FMD is an endothelium-dependent response to increased blood flow and wall stress. Increased stress on the vessel wall causes calcium-mediated potassium channels in the endothelial cell membrane to open, thereby increasing nitric oxide (NO) synthesis. Vasodilation occurs through endothelial NO production, which causes FMD. Impairment in vasodilation response (NO bioavailability) is a marker of ED and cardiovascular disease risk [13]. Patients with a low or impaired FMD response reportedly have a high risk of cardiovascular disease and a vascular phenotype prone to atherosclerosis [14]. In a healthy population, the normal FMD response range is 7–10% [15]. FMD is a non-invasive, validated method for identifying ED.

### 1.5. Nailfold Capillaroscopy

Capillaroscopy is a non-invasive imaging technique that provides information on microcirculation [16]. Using a capillaroscope, an image of the nail bed is obtained at 200× magnification. This imaging technique provides information on capillary density (number of capillaries in 1 mm), capillary size/morphology, and presence or absence of microhemorrhages [17]. A decrease in capillary density was defined as a reduction in the number of capillaries per unit area. Decreased renal capillary density increases vascular resistance and hypoxia, leading to the progression of renal disease [18]. In healthy adults, the normal capillary density is 7–12. Having fewer capillaries than this is defined as decreased capillary density or an avascular area [19].

In this study, we evaluated ultrasonographic measurement methods, such as CIMT, FMD, and NC, which have been previously used in the literature on this subject, in non-dialysis geriatric patients with CKD. We also investigated the relationship between these modalities and levels of sVE-cadherin and FGF23. In our study, we considered that sVE-cadherin could be an important potent biomarker of ED. We aimed to evaluate this marker alongside better-known modalities. Specifically, we included geriatric patients without known cardiovascular disease to examine this hypothesis in patients with potential risk. Evaluating the relationship between sVE-cadherin levels and GFR is a nuance of this study that has not been clearly demonstrated in the literature before. We hypothetically considered that sVE-cadherin levels might be further increased in cases of possible GFR decrease, and the potential positive effects of SGLT-2 inhibitors could be observed by influencing sVE-cadherin levels in the study subgroups.

## 2. Materials and Methods

The study included geriatric patients with CKD who were not receiving RRT and who presented to the Nephrology Outpatient Clinic of the Muğla Sıtkı Koçman University, Training and Research Hospital between 12 May 2023 and 11 September 2023. The inclusion criteria were as follows: patients with CKD who were not receiving RRT and aged 65 years and over. The exclusion criteria were as follows: age < 65 years; history of coronary artery disease, peripheral artery disease, active malignancy, or cerebrovascular events (ischemic/hemorrhagic); atrial fibrillation; heart failure; active immunosuppressive use; active infection, Raynaud’s phenomenon; and renal transplantation. All participants provided written informed consent to participate in accordance with the principles outlined in the Declaration of Helsinki. Ethical approval for this study was obtained from the Clinical Research Ethics Committee of Muğla Sıtkı Koçman University, dated 11 May 2023, with approval number E-72855364-050.01.04-609785 and decision number 11/XII. Registry-based monitoring was used to track the time of death.

### 2.1. Demographic Information, Laboratory Findings, Ultrasonographic and Capillary Measurements, and Biochemical Markers

Age, sex, height, weight, habits, comorbidities, and medication use were recorded in patients who met the inclusion criteria. Complete blood count, renal function tests, venous blood gas, HbA1c, parathyroid hormone (PTH), phosphorus, corrected calcium, albumin, serum lipids, 25-OH vitamin D, complete urinalysis, spot urine albumin/creatinine, and protein/creatinine tests were requested. The patients were staged according to their glomerular filtration rate (GFR) in accordance with the Kidney Disease: Improving Global Outcomes 2012 guidelines. Simultaneously, 5 mL of blood was drawn into a single plain tube and centrifuged at 1500–2000× *g* (RCF) for 10 min; the serum was separated and transferred to Eppendorf tubes. The serum in the Eppendorf tubes was stored at −80 °C until further analysis.

### 2.2. Ultrasonographic and Capillary Measurements

Using a General Electric Logiq 5 PRO (Milwaukee, WI, USA) ultrasound device, CIMT values were measured and recorded 1 cm below the right and left carotid artery bifurcation points in patients in the supine position. The arithmetic means of the right and left CIMT values were calculated and used for the evaluation. The basal brachial artery diameter (BAD) was measured from the non-dominant arm at a location where the anterior and posterior intimal surfaces were clearly visible. Blood pressure was measured in the non-dominant arm before the FMD was recorded. The cuff pressure was increased to 200 mmHg for those whose systolic blood pressure was below 200 mmHg to create occlusion, and patients were left in this position for 3 min. After 3 min, the cuff was deflated; the brachial artery diameter (endothelium-dependent vasodilator response [EDVR]) was measured again after 1 min. FMD values were recorded using the following formula: (EDVR − BAD)/BAD × 100.

Capillary imaging of the fourth finger of both hands was performed using a capillaroscopy device (Dino-Lite CapillaryScope x200 Pro, AnMo Electronics Corporation, Hsinchu City, Taiwan); thereafter, photographs were recorded. The patients were evaluated after resting at a temperature of 20–22 °C for at least 15 min. Subsequently, capillary density, morphology, morphological variations, bleeding, capillary diameter, giant capillaries, and avascular areas were evaluated using these photographs. The capillary size was measured from the apex of the capillary loop. A size of ±20 µm on average was defined as normal, between 20 µm and 50 µm was defined as dilated capillaries, and those that were morphologically normal but larger than 50 µm were defined as giant capillaries [20]. Determining capillary morphology, the European Rheumatology Society defined capillary morphology as normal and abnormal. Normal capillaries were considered to be straight, defined as a “hairpin,” or “tortious” (without folding) with curved afferent and efferent arms, or folded once or twice upon themselves; all other variations were defined as abnormal morphology. Microhemorrhages appeared red or brown due to extravasated erythrocytes and hemosiderin deposits resulting from damage to the capillary walls. These could be either single or multiple types. Focal microhemorrhages were observed in connective tissue diseases as well as conditions secondary to ED.

### 2.3. Biochemical Markers

Serum FGF23 (Cat# E0059Hu) and sVE-cadherin (Cat# E7006Hu) concentrations were measured by an enzyme-linked immunosorbent assay (ELISA) (Bioassay Technology Laboratory, Shanghai, China) according to the manufacturer’s instructions. FGF23 assay sensitivity was 2.49 pg/mL, with a range of 5–1500 pg/mL, and the inter-assay and intra-assay coefficients of variation were less than 10%. sVE-cadherin assay sensitivity was 0.096 ng/mL, with a range of 0.2–60 ng/mL, and the inter-assay and intra-assay coefficients of variation were less than 10%.

Measurements were taken using an ELISA plate reader (Multiskan GO microplate reader, Thermo Fisher Scientific, Waltham, MA, USA). The operating principle of ELISA is based on the biotin double-antibody sandwich technology. Samples were added to wells pre-coated with monoclonal antibodies before incubation. Biotin-labeled antibodies formed complexes with the streptavidin–HRP conjugate. After incubation, unbound conjugates were removed by washing. After adding the substrate solution, the resulting color intensity was measured at 450 nm, and the concentrations were calculated using a calibration graph plotted against the standards.

### 2.4. Statistical Analysis

The sample size was calculated using G*Power (v3.1.9.7) software. The type 1 error margin (alpha) and test power (1-beta) were set at 0.05 and 95%, respectively. Since there is no direct reference study in the literature comparing FGF23 and sVE-cadherin levels, a moderate effect size (r = 0.35) considered clinically significant was targeted. As a result of the analysis, the minimum sample size required to determine the correlation between the variables was calculated as *n* = 96. The study was completed with more participants than this number (*n* = 96) to maintain statistical power.

Data analysis was performed using R Statistical Software (version 4.5.3). Descriptive statistics for continuous variables are presented as mean ± standard deviation and minimum and maximum values. The normality of the variables was checked using the Shapiro–Wilk test. Since the number of groups was more than two, one-way analysis of variance (ANOVA) was applied to compare data with a normal distribution, and the Kruskal–Wallis test was applied to data that did not show a normal distribution.

Concurrent criterion-related validation was used to evaluate the consistency of FGF23 and sVE-cadherin measurements. The direction and strength of the relationship between the variables were analyzed using the Pearson correlation test when parametric test assumptions were met, and the Spearman Rank correlation test was used when assumptions were not met. A significance level of *p* < 0.05 was accepted for all statistical comparisons.

## 3. Results

This study included a total of 96 patients. The laboratory test results, comorbidities, and medications used are presented in Table 1 and Table 2.

The mean FMD values, serum FGF23 and sVE-cadherin levels, mean capillary measurements, and CIMT values of the patients are shown in Table 3.

**Figure 1:**
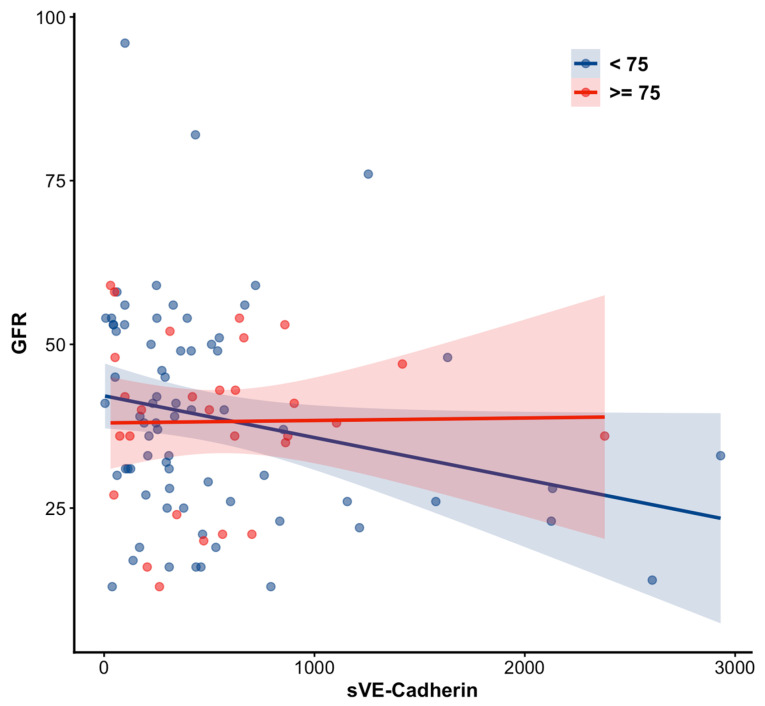
Comparison of GFR and sVE-cadherin levels between patients aged 75 and over and those under 75 years of age.

When comparing patients aged 75 and over with those under 75 years of age, no statistically significant differences were found between the two groups in terms of serum sVE-cadherin and FGF23 levels, mean CIMT, FMD, or capillary findings (Table 4). No significant difference was found between GFR and sVE-cadherin levels (Figure 1).

**Figure 2:**
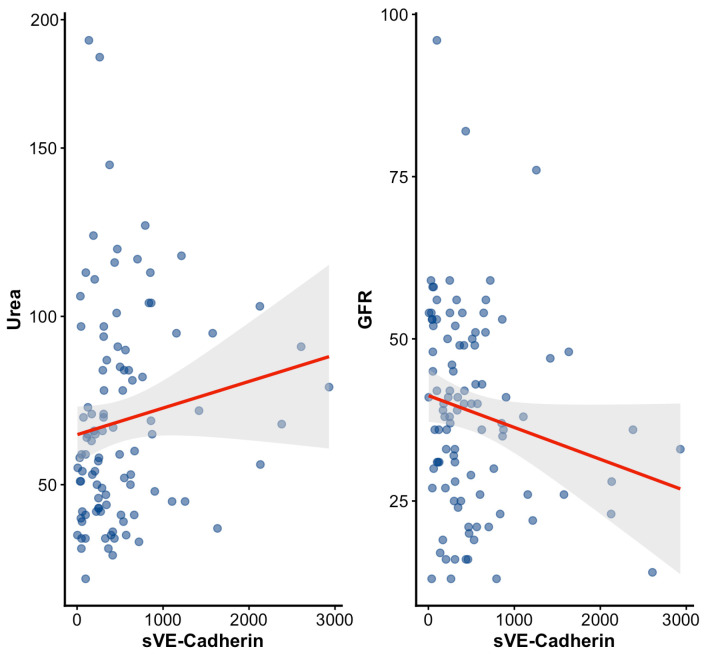
Correlation between serum sVE-cadherin levels and urea, GFR.

**Figure 3:**
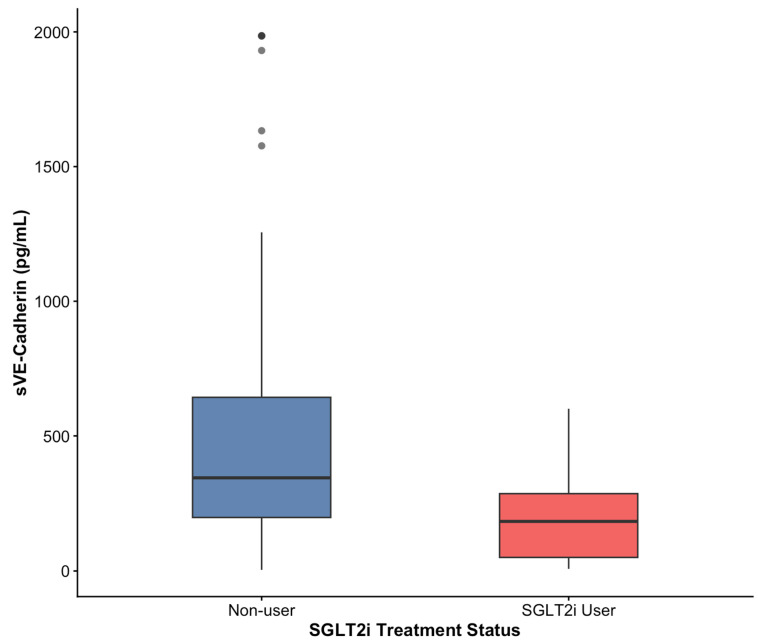
Serum sVE-cadherin levels were significantly lower in those using SGLT2is.

For correlation analyses, the Shapiro–Wilk test was used to determine whether the data were normally distributed. A significant negative correlation was found between serum sVE-cadherin levels and GFR (*p* < 0.05) (Figure 2). Similarly, a significant positive correlation was found between serum sVE-cadherin levels and serum urea concentrations (*p* < 0.05) (Figure 2). Serum sVE-cadherin levels tended to be higher but did not reach statistical significance (*p* = 0.077) in patients using calcium channel blockers (CCBs) and were significantly lower in those using sodium–glucose co-transporter2 inhibitors (SGLT2is) (*p* < 0.05) (Figure 3).

**Figure 4:**
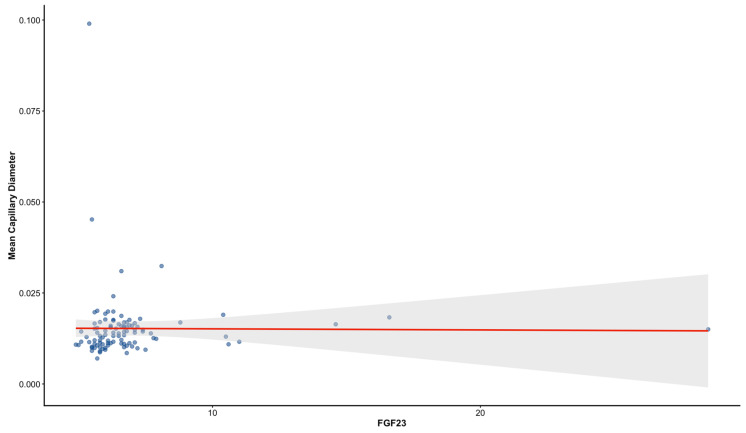
Correlation between serum FGF23 and mean capillary diameter.

A significant positive correlation was found between serum FGF23 and ferritin levels, neutrophil count, and mean diameter (*p* < 0.001, *p* < 0.05, and *p* < 0.05) (Figure 4). Serum FGF23 levels were significantly higher in patients with hypertension (HT) and those using CCB (*p* < 0.05; *p* < 0.05).

**Figure 5:**
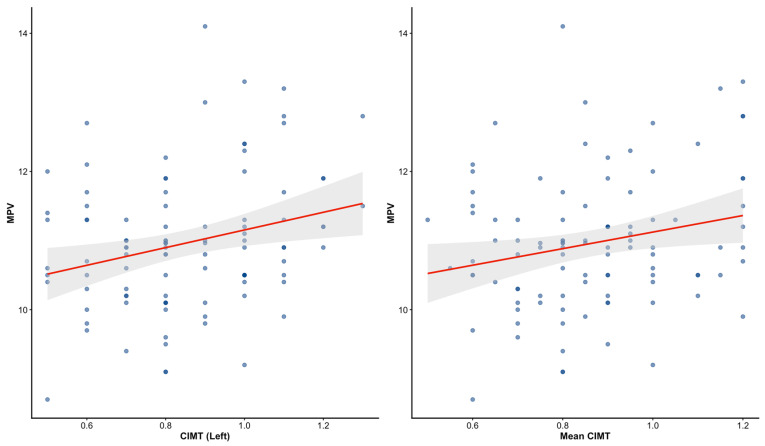
Correlation between the left and mean CIMT values and MPV.

A significant positive correlation was found between the left and mean CIMT values and mean platelet volume (MPV) (*p* < 0.05; *p* < 0.05) (Figure 5).

A significant positive correlation was found between the FMD and serum ferritin levels (*p* < 0.05). A significant negative correlation was found between FMD measurements and the neutrophil count and neutrophil/lymphocyte ratio (*p* < 0.05; *p* < 0.05). The FMD was significantly higher in women than in men (*p* < 0.05).

A significant positive correlation was found between a decreased mean capillary count and low GFR (*p* < 0.05); a significant negative correlation was found between a decreased mean capillary count and increased PTH levels (*p* < 0.05). The mean capillary count was significantly higher in patients using dipeptidyl peptidase 4 inhibitors (*p* < 0.05). A negative correlation was found between the mean capillary diameter and uric acid/albumin, CRP/albumin, CRP, serum phosphorus level, serum urea, and CKD stage (*p* < 0.05, *p* < 0.05, *p* < 0.05, *p* < 0.05, *p* < 0.05, and *p* < 0.05, respectively).

**Figure 6:**
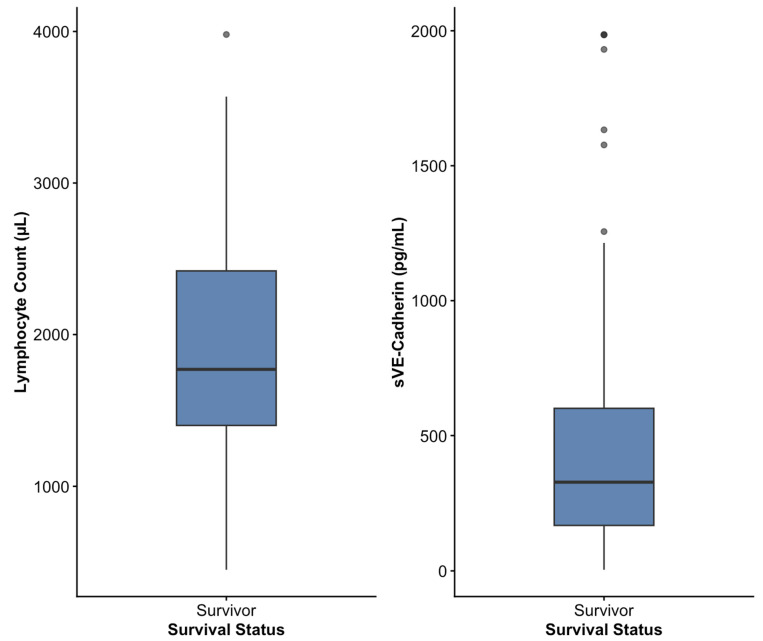
The lymphocyte count was higher in survivors; although this was not statistically significant.

After 2.3 years of patient follow-up, nine patients passed away. Comparing the data of those who died and those who survived, the lymphocyte count was higher in survivors; however, this was not statistically significant (*p* = 0.08) (Table 5, Figure 6). Despite this, the mean basal sVE-cadherin levels were higher in patients who died (Table 5, Figure 6).

The results adjusted for CKD stage and confounding factors are shown in Table 6 and Table 7. Multiple regression analyses have also been added.

## 4. Discussion

Endothelial stability relies on intact adherence junctions between endothelial cells. Structural modifications of VE-cadherin, which occur through phosphorylation of intracellular tyrosine residues or detachment of the extracellular domain, play a role in tumor necrosis factor-alpha (TNF-α) mediated by microvascular permeability [21]. Loss of permeability control leads to various pathological conditions, including hypoxia, chronic inflammatory diseases, and atherosclerosis. ED and increased permeability could cause atherosclerosis, a chronic inflammatory condition characterized by lipid-rich plaque accumulation in vessel walls. In an experimental study by Sutton et al., 24 h after renal ischemia, the loss of staining for VE-cadherin in a large portion of the renal microvasculature suggested disruption to the normal junction complex between endothelial cells of renal microvasculature. Changes in the actin cytoskeleton of renal microvascular endothelial cells occurred before changes in VE-cadherin staining at intercellular junctions. These findings are consistent with the mechanism by which the loss of integrity in the actin cytoskeleton contributes to the disruption of actin-associated adhesion junctions and concomitant permeability defects [22].

In our study, we found a significant negative correlation between serum sVE-cadherin levels and GFR in geriatric non-dialysis patients with CKD. Similarly, a significant positive correlation was found between serum sVE-cadherin levels and urea concentrations. This inverse correlation with GFR suggests that decreased renal function and an inflammatory environment can increase serum sVE-cadherin levels. In a study including patients with AKI, sVE-cadherin levels were higher in patients with severe AKI due to sepsis than in those with mild AKI [23]. In our study, sVE-cadherin levels were higher in patients with HT. ED is an important mechanism in the development of HT and is an early indicator of various cardiovascular diseases. The damaging effect of Ang II on sVE-cadherin was demonstrated in a model of Ang II-induced damage to hypertensive endothelial cells [24]. We also found higher sVE-cadherin levels in patients with HT, which supports this finding.

In a prospective follow-up study including 228 patients diagnosed with sepsis in the intensive care unit by Yu et al., 80 patients developed AKI requiring RRT; serum sVE-cadherin levels were significantly higher in these patients than in those who did not develop AKI. Furthermore, these patients had significantly longer hospital stays, stays in the intensive care unit, mechanical ventilation duration, and higher mortality rates [25]. AKI, which is one of the leading causes of organ failure in patients with sepsis, is associated with decreased renal blood flow and ED [26]. In a previous study, the high Acute Physiological and Chronic Health score was used to indicate sepsis severity, and high serum sVE-cadherin levels in patients requiring RRT suggested that sVE-cadherin is associated with ED.

In our study, serum sVE-cadherin levels were significantly lower in patients treated with SGLT2i. Experimental studies have shown that increased stress on cells increases ROS. This promotes the internalization and degradation of VE-cadherin, the main component of adhesive junctions between endothelial cells, leading to impaired endothelial barrier function, increased permeability, and decreased NO bioavailability [27]. SGLT2is, on the other hand, have been shown to increase NO bioavailability [28]. An experimental study showed that the loss of VE-cadherin due to stress applied to human coronary artery endothelial cells (10% stretch-induced ROS production) was reduced by SGLT2is [29]. Supporting studies have evidenced the negative impact of 25-hydroxycholesterol (25OHC) detection in atherosclerotic plaques on endothelial barrier function, showing that the loss of VE-cadherin due to 25OHC was reduced by SGLT2is [30].

In patients with diabetes mellitus, a persistent inflammatory microenvironment damages endothelial cells and facilitates the development of cardiovascular diseases. Inflammation-induced endothelial barrier dysfunction is the first step in the development of vascular disease. TNF-α is known to disrupt endothelial cell–cell junctions and increase vascular permeability. Empagliflozin directly reduces ROS production and restores NO bioavailability in endothelial cells stimulated by the pro-inflammatory cytokine TNF-α, which exerts a potential cardiac damage-preventive effect [31]. It has been thought that the reduction in intracellular Ca^2+^ by empagliflozin is one of the key mechanisms underlying its antioxidative effect. Li et al. suggested that empagliflozin inhibited protein kinase C activity by reducing intracellular Ca^2+^, thus preventing NADPH oxidase activation and ROS formation in endothelial cells subjected to enhanced stretching. These data show that Na^+^/H^+^ exchanger 1 plays an important role in the antioxidant effects of empagliflozin on human endothelial cells [32]. Similarly, dapagliflozin mitigates oxidative stress-induced ED by activating sirtuin 1 in human umbilical vein endothelial cells [33].

Oscillatory shear stress disrupts endothelial barrier integrity by increasing cytoskeletal tension and enhancing the phosphorylation of VE-cadherin, a key component of endothelial adhesion junctions. The necessity of VE-cadherin for eNOS activation during shear stress regulation has been previously demonstrated [34]. Another toxic agent, “intermittent hypoxia,” has been shown to cause VE-cadherin degradation in vivo in experimental studies (C57BL/6J mice) [35]. SGLT2is might have a protective effect against this degradation through its anti-hypoxic effect. It is suggested that SGLT2is also have positive effects on erythropoiesis and angiogenesis through stimulation of the hypoxia-inducible factor pathway.

In a prospective study including 96 patients with CKD who did not receive RRT, patients were grouped according to CKD stage (stages 2–4). Measurements were taken of basal capillary density, density of the capillary filling phase after arterial occlusion, and capillary density after venous congestion. In all three phases, capillary density decreased as the CKD stage increased. In addition, ultrasonographic CIMT values increased as the CKD stage increased; however, the difference was not statistically significant. Although the number of patients in each group was equal in this study, patients with early stages were younger [36]. The significant positive correlation found between the mean capillary number and GFR in our study was consistent with this. Similarly, in a study by Schoina et al., the mean capillary number decreased as the GFR decreased, and an inverse correlation was found with PTH. Using multivariate linear regression analysis, reduced estimated GFR (eGFR), presence of diabetes mellitus, and increased PTH levels were independently and inversely associated with functional capillary density [36].

In patients with CKD, a decrease in microvascular structures and marked structural changes involving heterogeneous and widely avascular areas were observed, including the capillaries, small arterioles, and venules. It has also been shown that the microvascular density in the heart muscles of the same animals decreases in parallel, demonstrating the systemic “toxic” effect of uremia on microcirculation [37].

CKD (uremic angiopathy) causes systemic microvascular rarefaction. Heterogeneous loss of the microvascular network structure and presence of avascular patches result in decreased organ perfusion, shunting, and tissue hypoxia. In experimental CKD models, microvascular loss precedes fibrosis; furthermore, the progression of fibrosis is closely related to rarefaction. Therefore, uremic microangiopathy and chronic inflammation are important factors in tissue fibrosis. Microvascular disease is a major pathogenic factor for the progression of CKD and development of widespread severe organ dysfunction and multiple morbidities that occur as the disease progresses. In a study including patients with diabetes, the capillary diameters at both terminals were significantly diminished in the reduced eGFR group (*p* < 0.0001). The group with an eGFR < 30 mL/min had fewer capillaries (*p* = 0.0061) and smaller capillary diameters (*p* < 0.0001) than the other groups. Renal hypoxia is a significant catalyst for the progression of diabetic nephropathy and CKD. An impaired oxygen supply leads to a decrease in the number of capillaries, tubular dedifferentiation, inflammatory reactions, and fibrosis. These changes are reflected in microvascular abnormalities detectable by nailfold capillaroscopy [38]. In our study, although the mean age was higher and those with cardiac disease were excluded, the mean capillary diameter decreased as the CKD stage increased. In addition, the mean capillary number decreased as the GFR decreased, in a manner similar to that in this study.

In the present study, the average number of capillaries was higher when using dipeptidyl peptidase-4 (DPP4). Pharmacologically, DPP4 inhibitors are known to reduce EndoMT and renal fibrosis in patients with diabetic nephropathy. Linagliptin inhibits EndoMT by blocking TGFβ and AGE-RAGE signaling in endothelial cells, which are known pathways of EndoMT activation. EndoMT is also significantly associated with loss of functional and structural integrity, including vascular reduction, increased vascular permeability, and interstitial fibrosis. In the vascular system, DPP4 is expressed by endothelial and smooth muscle cells; its inhibition has been associated with the restoration of endothelium-dependent relaxation by reversing ED. DPP4i increases NO bioavailability. In patients with type 2 diabetes mellitus, a decrease in FMD is observed, and glycemic control is associated with increased DPP4 activity, independent of HT, lipid profile, and systemic inflammation [39]. Studies have shown that DPP4is can slow or reverse vascular aging via various pathways. These effects are reported to be mediated by correcting endothelial cell dysfunction (reducing TNF-α, NF-kB pathway, and oxidative stress), reducing cell senescence/apoptosis (regulating AMPK/SIRT1/Nrf2 signaling pathway, and modulating PKA signaling), enabling endothelial progenitor cell function, number, and mobilization (activating AMPK/ULK1 signaling pathway, via SDF-1a/CXCR4 axis) [40]. We believe that the higher mean capillary number observed in patients taking DPP-4 inhibitors may be secondary to positive endothelial effects. The fact that this positive effect can also be observed in geriatric patients is an important inference for this age group.

In an experimental study using microcomputed tomography, Ehling et al. demonstrated that advanced kidney disease and fibrosis caused a reduction in vessel diameters [41]. In our study, a negative correlation was found between mean capillary diameter and uric acid/albumin and CRP/albumin ratios, and between CRP, serum phosphorus level, urea, and CKD stage. Similarly, in the comprehensive study by Hsu et al., including diabetic patients, it was shown that a decrease in capillary diameter correlated with a decrease in GFR [38]. Capillary diameter was significantly lower in patients with primary glomerular diseases than in healthy controls; although not statistically significant, capillary density was lower in those with primary glomerular diseases than in healthy individuals [42].

MPV is not only an indicator of platelet mass, but also an entity that expresses its activation. MPV has been shown to reflect inflammatory load and disease activity in various chronic inflammatory diseases. In newly diagnosed patients with HT (mean creatinine 0.72 ± 0.56 mg/dL), MPV showed a moderate correlation with CIMT (r = 0.60, *p* = 0.0001) [43]. In another study, univariate analysis of data from patients who experienced atherothrombotic stroke showed positive correlations between MPV and the degree of carotid atherosclerosis (*p* < 0.00007) and CIMT (*p* < 0.00002). When the MPV was greater than 11.25 fl, a significant correlation was found with the severity of carotid stenosis (OR: 2.9, *p* < 0.00007) [44]. In the study by Ju et al., patients with CKD (mean age 62.41 ± 13.59 years) or CVD had higher MPVs than patients without CAD or CVD [45]. In our study, we also observed a significant positive correlation between the increase in mean CIMT values and MPV. We suggest that this is an important parameter for predicting cardiovascular and cerebrovascular risks in geriatric patients with CKD.

In our study, the relationship between FGF23 and ED and inflammation was evaluated and found to be positively correlated with serum ferritin levels and mean and right-hand capillary diameters; however, we could not demonstrate a correlation with FGF23-FMD in our study. In a study by Natsuki et al., this relationship was not observed in patients with type 2 diabetes mellitus; instead, it was emphasized that chronic hyperglycemia could be the cause. However, it has also been suggested that age and other comorbidities could easily affect this correlation [46]. Our patient population was entirely geriatric, which led us to suggest that “inflammaging” might have obscured the results. We suggest that studies comparing geriatric and non-geriatric groups could answer this question. The relationship between FGF23 and arterial thickness and calcification can change not only in patients with CKD, but also with increasing age. High serum FGF23 levels are associated with adverse cardiac outcomes such as left ventricular hypertrophy, cardiac fibrosis, and HT. They are also independently associated with increased all-cause and cardiovascular mortalities in patients with CKD. In our study, we found that the FGF23 levels were significantly higher in patients with HT. The ARIC study showed that approximately 27% of adults aged 45–64 years developed HT after a 6-year follow-up, and the prevalence was higher in the group with the highest decimal percentile of baseline serum FGF23 levels, independent of renal function [47].

The neutrophil-to-lymphocyte ratio (NLR) is considered an indirect marker of ED. Solak et al. demonstrated an inverse relationship between the NLR and FMD in patients with stage 3–5 CKD [48]. The results of our study support this relationship.

Our findings demonstrate that uremia could enhance endothelial damage by altering intercellular connections. Uremic toxins could trigger active free radicals, endothelial microparticle production, and cytoskeletal remodeling, while interfering with intercellular connections by increasing permeability. In patients with uremia, these cellular connections could be disrupted, promoting endothelial damage and progressive cardiovascular disease. In a study in which immunolabeling was performed on iliac renal arteries from donors (control group) and recipients with CKD, intact and continuous endothelium with strong VE-cadherin was observed in the arterial sections of donors, whereas in the arterial sections of the recipients, a decrease in VE-cadherin immunolabeling, endothelial damage, and disruption in the endothelial cell monolayer structure, characteristic of structural damage, were observed. The loss of endothelial barrier integrity is due to the disruption of intercellular connections. The uremic environment affects endothelial cell adhesion junctions and VE-cadherin expression [49].

Chang et al. have shown that vascular aging (with the potential for increased Src activity) causes thinning and deterioration in arterial endothelial adhesion junctions. This is hypothesized to be related to increased VE-cadherin phosphorylation, the loss of VE-cadherin from adhesion junctions, and endothelial dysfunction in aged arteries [50]. Therefore, research on whether VE-cadherin damage in geriatric patients with CKD could be solely related to CKD and the possible contribution of age is warranted.

Our study has several limitations. The fact that this was a single-center study and a limited number of patients were included is a significant shortcoming. This might have been due to our very strict exclusion criteria for geriatric patients with CKD. Specifically, the exclusion of patients with known coronary artery disease was a contributing factor. In our study, we attempted to analyze a group of geriatric patients with very limited medical history. Our main goal was to perform endothelial function assessments in a more specific, relatively “healthy” group of patients, specifically excluding patients with known cardiovascular disease from the study population. We wanted to test the validity of a proactive approach in geriatric CKD patients with no history of cardiovascular disease who may also have high endothelial risk factors and already have a high risk of mortality. In this study, we included geriatric patients as assessing frailty could also be important. The absence of a control group, use of an observational design, possible residual confounding, multiple testing, small medication subgroups, limited event counts, and an inability to infer causality were among the other limitations. Without a healthy control group, we cannot determine whether sVE-cadherin levels are elevated in CKD compared to age-matched controls without CKD. Moreover, since long-term follow-ups of patients could be conducted in different locations, information on mortality could only be obtained from a central information system; therefore, precise data on the exact causes of death was not available. However, although the study was underpowered for this endpoint, we believe that sharing mortality data after a not-so-short observation period may be important. In particular, we believe this study is valuable for future studies, as it shows an association between sVE-cadherin and mortality for the first time in the literature. Finally, the lack of other studies examining sVE-cadherin levels in non-dialysis geriatric patients with CKD made comparisons difficult in some cases.

## 5. Conclusions

The search for a better understanding of ED and, consequently, non-traditional cardiovascular risk factors in patients with CKD continues. At the guideline level, rational markers clearly surpass their significant horizon. In this comprehensive study, we aimed to present a different perspective, particularly for CKD patients in the geriatric age range, as this age group has a high concentration of CKD. To the best of our knowledge, there are no studies in the literature on sVE-cadherin levels in geriatric patients with CKD; thus, our study has unique value in this field. We showed, for the first time, a relationship between low GFR and high sVE-cadherin levels. As such, our study will pave the way for further research on the utility of sVE-cadherin as a diagnostic or prognostic factor for ED in patients with CKD.

## Figures and Tables

**Table 1:** Laboratory Test Results.

Laboratory Parameter	Mean ± SD	Median (Min–Max)
HbA1c (%)	6.15 ± 1.06	5.8 (4.6–11.1)
Urea (mg/dL)	68.41 ± 31.98	59.5 (22–182)
Creatinine (mg/dL)	1.74 ± 0.73	1.51 (0.74–4.3)
GFR (ml/min/1.73 m^2^)	39.34 ± 15.32	39.5 (13–96)
Sodium (mmol/L)	140.08 ± 3.55	140.9 (121–147)
Potassium (mmol/L)	4.74 ± 0.55	4.8 (3.11–6.07)
Albumin (g/dL)	4.43 ± 0.33	4.45 (3.5–5.1)
Uric acid (mg/dL)	6.81 ± 1.86	6.5 (3.1–12.5)
Corrected Ca (mg/dL)	9.5 ± 0.56	9.5 (7.86–11.58)
Phosphorus (mg/dL)	3.6 ± 0.7	3.58 (1.5–6.1)
Magnesium (mg/dL)	2.05 ± 0.31	2.07 (1–2.75)
Triglyceride (mg/dL)	144.61 ± 73.61	127.5 (55.9–485.6)
HDL (mg/dL)	50.39 ± 14.07	48.4 (25.1–105)
LDL (mg/dL)	108.23 ± 34.08	106.75 (38.9–188.5)
uACR (mg/g)	293.98 ± 557.46	63 (1–3480)
uPCR (mg/g)	496.59 ± 643.16	242.5 (54–3646)
Ferritin (ng/mL)	99.23 ± 129.03	61.9 (8.55–781)
Parathormone (pg/mL)	95.13 ± 76.36	68.25 (5.86–402)
pH	7.36 ± 0.05	7.36 (7.23–7.48)
Venous bicarbonate (mEq/L)	24.56 ± 3.56	24.8 (15.9–33.1)
Hemoglobin (g/dL)	12.14 ± 1.8	12.25 (7.8–15.9)
Thrombocyte (µL)	248,562.5 ± 68,721.38	243,000 (90,000–489,000)
MPV (fL)	10.96 ± 1.02	10.9 (8.7–14.1)
Neutrophile (µL)	4575.42 ± 1632.69	4490 (1640–9590)
Lymphocyte (µL)	1924.9 ± 717.92	1765 (450–3980)
CRP (mg/L)	5.24 ± 9.75	2.34 (0.6–72)
25 (OH) vit D (ng/mL)	22.67 ± 12.96	21.8 (3–75.3)

**Table 2:** Comorbidities and medications.

Comorbidity/Medication	*n*	%
DM	50	52.0833333
HT	89	92.7083333
RAAS Blocker	73	76.0416667
Beta Blocker	34	35.4166667
Ca channel blocker	39	40.625
Alpha Blocker	20	20.8333333
Diuretic	59	61.4583333
Metformin	14	14.5833333
SGLT2i	13	13.5416667
DPP4i	33	34.375
Statin	22	22.9166667
Insulin	17	17.7083333

**Table 3:** Mean-median values of FMD, serum FGF23 and sVE-cadherin levels, NC measurements, and CIMT.

Vascular/Capillary Parameter	Mean ± SD	Median (Min–Max)
Right CIMT (cm)	0.88 ± 0.21	0.8 (0.5–1.4)
Left CIMT (cm)	0.85 ± 0.2	0.8 (0.5–1.3)
Mean CIMT (cm)	0.86 ± 0.19	0.85 (0.5–1.2)
FMD (%)	12.24 ± 6.88	9.41 (3.23–30.43)
Right-hand capillary count (*n*)	7.39 ± 0.65	7 (6–9)
Right-hand capillary diameter (μm)	14.42 ± 5.83	13.2 (6.1–40.5)
Right-hand capillary length (μm)	180 ± 60	170 (60–480)
Left-hand capillary count (*n*)	7.32 ± 0.69	7 (5–9)
Left-hand capillary diameter (μm)	14.55 ± 5.82	13.8 (6.5–57.9)
Left-hand capillary length (μm)	170 ± 60	170 (70–370)
Mean capillary count (*n*)	7.5 ± 0.68	7 (6–9)
Mean capillary diameter (μm)	15.41 ± 10.06	14.1 (7–99)
Mean capillary length (μm)	170 ± 50	170 (70–420)
sVE-cadherin (pg/mL)	416.8 ± 355.3	311.9 (4.08–1633)
FGF23 (pg/mL)	6.89 ± 2.82	6.3 (4.9–28.5)

**Table 4:** Comparison of patients aged 75 and over with those under 75 years of age, in terms of serum sVE-cadherin and FGF23 levels, mean CIMT, FMD, or capillary findings.

Variable	Distribution Type	Shapiro–Wilk *p*	Uygulanan Test	Raw *p*	FDR/BH
CIMTright	Non-Parametric (Skewed)	0.0002	Mann–Whitney U	0.4215	0.8841
CIMTleft	Non-Parametric (Skewed)	0.0042	Mann–Whitney U	0.7073	0.8841
CIMTmed	Non-Parametric (Skewed)	0.0021	Mann–Whitney U	0.4782	0.8841
FMD	Non-Parametric (Skewed)	0	Mann–Whitney U	0.6517	0.8841
Rcount	Non-Parametric (Skewed)	0	Mann–Whitney U	0.1736	0.8841
Rdiam	Non-Parametric (Skewed)	0	Mann–Whitney U	0.891	0.9069
Rlength	Non-Parametric (Skewed)	0	Mann–Whitney U	0.7964	0.9069
Lcount	Non-Parametric (Skewed)	0	Mann–Whitney U	0.2582	0.8841
Ldiam	Non-Parametric (Skewed)	0	Mann–Whitney U	0.3817	0.8841
Llength	Non-Parametric (Skewed)	0.0007	Mann–Whitney U	0.6116	0.8841
Meancount	Non-Parametric (Skewed)	0	Mann–Whitney U	0.2055	0.8841
Meandiam	Non-Parametric (Skewed)	0	Mann–Whitney U	0.6899	0.8841
Meanlength	Non-Parametric (Skewed)	0.0002	Mann–Whitney U	0.6575	0.8841
sVE-Cadherin	Non-Parametric (Skewed)	0	Mann–Whitney U	0.1525	0.8841
FGF23	Non-Parametric (Skewed)	0	Mann–Whitney U	0.9069	0.9069

**Table 5:** Comparision of the data of those who died and those who survived.

Variable	Exitus (*n* = 9)	Survivor (*n* = 87)	*p*	Test
sVE Cadherin (pg/mL)	312.9 (248.7–643.3)	309.3 (131.6–548.4)	0.466	Mann–Whitney U
FGF-23 (pg/mL)	6.3 (5.9–6.9)	6.3 (5.75–6.8)	0.5334	Mann–Whitney U
CIMT median (cm)	0.85 (0.75–1.1)	0.85 (0.7–1)	0.82	Mann–Whitney U
FMD (%)	16.98 (5.13–17.86)	9.3 (6.25–17.82)	0.7014	Mann–Whitney U
Neutrophil (µL)	4350 (3160–4740)	4520 (3550–5725)	0.5093	Mann–Whitney U
Lymphocyte (µL)	1500 ± 707.21	1968.85 ± 708.5	0.0884	*t*-Test
N/L ratio	3.2 (2.77–4.44)	2.27 (1.56–3.33)	0.1448	Mann–Whitney U
Vit D (ng/mL)	23.5 (17.75–26.3)	21.8 (13.75–28.65)	>0.99	Mann–Whitney U

**Table 6:** The results adjusted for CKD stage and confounding factors.

Variable	Distribution Type	Shapiro *p*	Test	Raw *p*	Adjusted *p* (FDR)
sVECadherin	Non-Parametric	0	Kruskal–Wallis	0.33071337	0.385832262
FGF23	Non-Parametric	0	Kruskal–Wallis	0.75393728	0.753937281
GFR	Non-Parametric	0.0043	Kruskal–Wallis	1.1022 × 10^−17^	1.5431 × 10^−16^
creatinine	Non-Parametric	0	Kruskal–Wallis	1.3264 × 10^−14^	9.28492 × 10^−14^
urea	Non-Parametric	0	Kruskal–Wallis	7.833 × 10^−11^	3.6554 × 10^−10^
HbA1c	Non-Parametric	0	Kruskal–Wallis	0.16022466	0.268324284
albumin	Non-Parametric	0.0177	Kruskal–Wallis	0.16267961	0.268324284
CRP	Non-Parametric	0	Kruskal–Wallis	0.31731744	0.385832262
Dvit	Non-Parametric	0	Kruskal–Wallis	0.08732506	0.24451016
CIMTmed	Non-Parametric	0.0021	Kruskal–Wallis	0.17249418	0.268324284
FMD	Non-Parametric	0	Kruskal–Wallis	0.21883526	0.306369358
Medcount	Non-Parametric	0	Kruskal–Wallis	0.11737242	0.268324284
Meddiam	Non-Parametric	0	Kruskal–Wallis	0.03610909	0.126381799
Medlength	Non-Parametric	0.0002	Kruskal–Wallis	0.4429233	0.476994321

**Table 7:** Multiple regression.

Model	Predictor	Beta	S.Error	t	*p*
Model 1: sVE Cadherin	(Intercept)	150.44352	541.922612	0.27761071	0.78196236
Model 1: sVE Cadherin	Age	1.18689528	6.80584053	0.17439364	0.8619567
Model 1: sVE Cadherin	DM	99.4073825	80.5949128	1.23342006	0.22070349
Model 1: sVE Cadherin	HT	52.920919	145.661091	0.36331541	0.71724059
Model 1: sVE Cadherin	GFR	1.11623334	2.55319294	0.43719114	0.66304374
Model 1: sVE Cadherin	SGLT2	−229.780089	118.059229	−1.94631194	0.05480778
Model 1: sVE Cadherin	CCB	142.461855	79.4931837	1.79212668	0.07654953
Model 1: sVE Cadherin	Meddiam	0.5355408	3.64866149	0.14677733	0.88364352
Model 2: FGF23	(Intercept)	2.14614078	4.38094001	0.48988134	0.62542326
Model 2: FGF23	Age	0.0424567	0.05473827	0.77563098	0.44002306
Model 2: FGF23	DM	−0.40623348	0.60614731	−0.67018937	0.50447235
Model 2: FGF23	HT	1.13448688	1.18596225	0.95659612	0.34136241
Model 2: FGF23	GFR	0.01353921	0.0205494	0.65886146	0.51168575
Model 2: FGF23	CCB	0.63030063	0.64755257	0.97335824	0.33301339
Model 2: FGF23	Meddiam	0.00130243	0.0296976	0.04385631	0.96511719
Model 3: Meddiam	(Intercept)	33.5907505	16.7895704	2.00069149	0.04854337
Model 3: Meddiam	Age	−0.24741335	0.20096875	−1.23110355	0.22160189
Model 3: Meddiam	DM	−0.81649248	2.21879309	−0.36798947	0.71377489
Model 3: Meddiam	HT	1.63195157	4.32746335	0.37711505	0.70700642
Model 3: Meddiam	GFR	0.01356193	0.10224686	0.13263914	0.89478508
Model 3: Meddiam	CRP	−0.03705128	0.11418902	−0.32447324	0.74635865
Model 3: Meddiam	Urea	−0.02333922	0.04912366	−0.47511158	0.63589806
Model 3: Meddiam	sVE Cadherin	−5.3328 × 10^−5^	0.00309686	−0.01722007	0.98630048
Model 3: Meddiam	FGF23	0.00901779	0.38922743	0.02316844	0.98156897

## Data Availability

The original contributions presented in this study are included in the article. Further inquiries can be directed to the corresponding author.
